# Mycobacterium Genavense Granuloma Mimicking a Brain Tumor: A Case Report

**DOI:** 10.7759/cureus.1547

**Published:** 2017-08-07

**Authors:** Atrin Toussi, Amir Goodarzi, Edwin Kulubya, Darrin J Lee, Ben Waldau

**Affiliations:** 1 Neurological Surgery, University of California, Davis Medical Center

**Keywords:** mycobacterium, genavense, intracranial, hiv, aids, tumor

## Abstract

Mycobacterium genavense (M. genavense) is a rare, non-tuberculous organism that commonly leads to gastrointestinal infections in immunocompromised patients. Only two cases of intracranial M. genavense infection have been reported to date. We describe a third case of M. genavense granuloma mimicking a right parietal intracranial mass, and review the literature on this exceedingly rare pathology.

## Introduction

 Mycobacterium genavense (M. genavense) is a rare, opportunistic, non-tuberculous mycobacterium that can cause significant morbidity and mortality in immunocompromised patients. This organism is frequently discovered in patients who carry the diagnosis of the human immunodeficiency virus (HIV), especially in the setting of an absolute CD4 count below 100. M. genavense can also cause infections in organ transplant recipients, patients on high-dose steroids, and those with other immunodeficiency syndromes. M. genavense infections are commonly located in the liver, bone marrow, small intestines, spleen, lungs, and lymph nodes. However, M. genavense infections of the central nervous system (CNS) are exceedingly rare [1]. The authors present a case of an incidentally identified intracranial mass, initially concerning for a neoplasm, that was ultimately identified as a M. genavense infection. Only two previously reported cases of intracranial infection with M. genavense exist and we review the literature on this rare pathology. 

## Case presentation

A 39-year-old woman with a past medical history of recurrent sinusitis, intermittent blurry vision, generalized fatigue, and unintentional weight loss presented for evaluation of a right parietal brain mass. At the time of presentation, she denied fevers, chills, cough, rhinorrhea, or dermatologic abnormalities.

**Figure 1:**
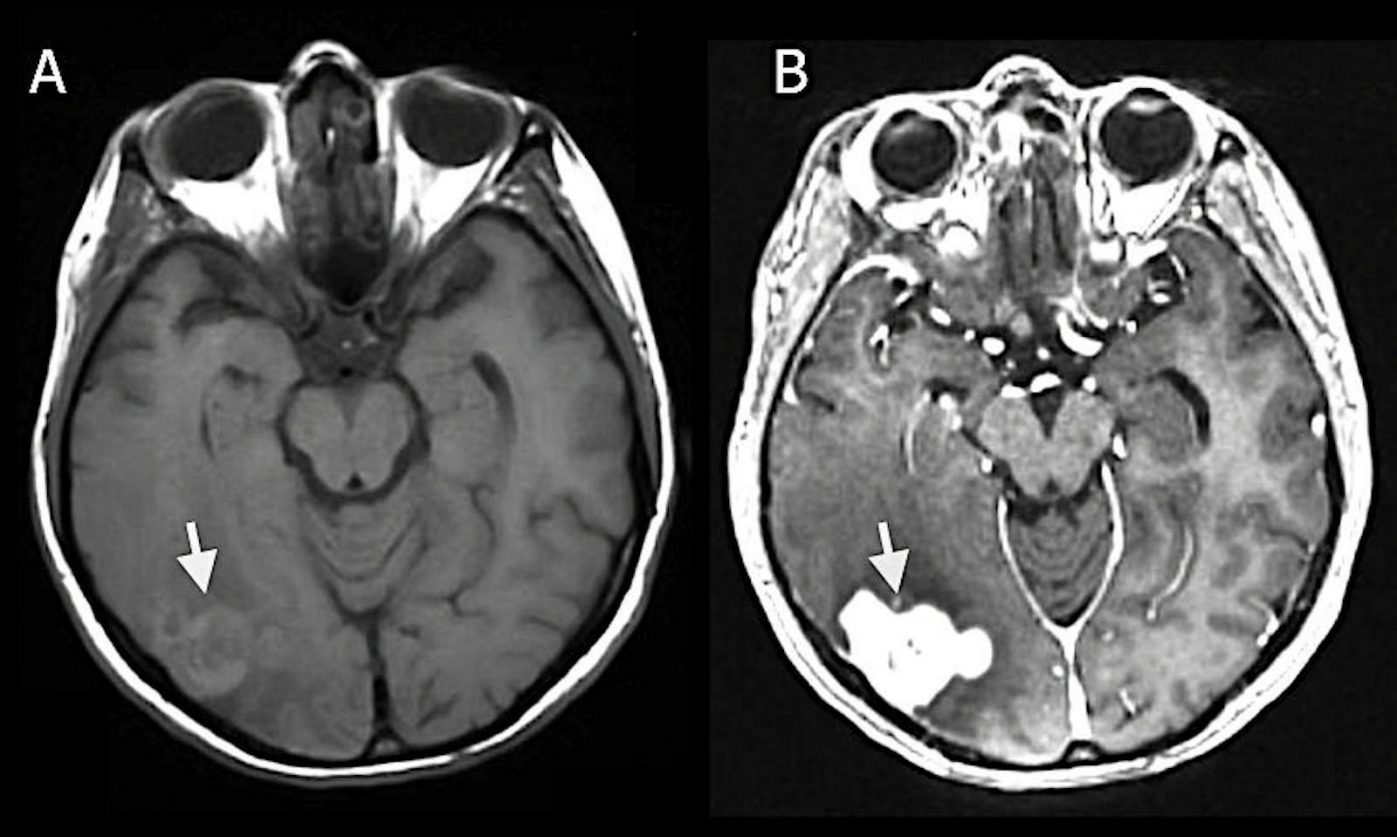
Magnetic resonance imaging T-1 weighted image (MRI T1WI) MRI T1WI Pre (A) and Post (B) gadolinium axial sequences demonstrating a 2 cm x 3 cm, avidly enhancing, lobular, right-parietal mass with leptomeningeal involvement (white arrow).

**Figure 2:**
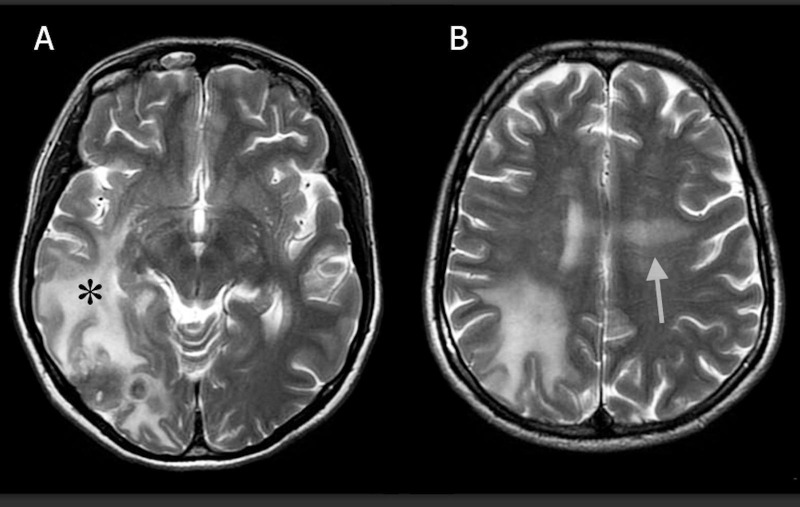
Magnetic resonance imaging T-2 weighted image (MRI T2WI) (A) MRI T2WI axial sequence demonstrates extensive perilesional edema extending anteriorly and inferiorly into the right temporal lobe indicated by the black asterisk. (B) MRI T2WI image demonstrates bifrontal subcortical edema extending across the body of the corpus callosum indicated by the white arrow.

On physical exam, the patient was neurologically intact and her visual fields were full. A computed tomography (CT) scan of her head identified a hypodense right parietal intracranial mass. The magnetic resonance imaging (MRI) of the brain demonstrated a 2 cm x 3 cm, avidly enhancing, lobular, right parietal mass with involvement of both the adjacent dura and pia mater (Figure 1A-1B). There was significant perilesional edema extending to the temporal and parietal lobes in the right hemisphere (Figure 2A). Concurrently, a non-enhancing, T2 hyperintense lesion was noted within the bilateral subcortical white matter of the frontal lobes as well as the body of the corpus callosum (Figure 2B). There was no associated diffusion restriction in either of the lesions.

Metastatic workup via CT of the chest, abdomen, and pelvis was positive for a nonspecific 4 mm left upper lobe nodule without aggressive features.

On initial presentation, the patient was accompanied by her boyfriend and denied any previous exposure to intravenous drug use or high-risk sexual behavior. The patient’s preoperative labs were significant for a low white blood cell (WBC) count of 2.8 K/MM3 with an absolute neutrophil count of 1.4 K/MM3 and an absolute lymphocyte count of 0.6 K/MM3. Furthermore, urinalysis results indicated a urinary tract infection.

Based on the available imaging, the preoperative differential diagnosis was broad and included a lymphoma, metastatic tumor, atypical meningioma, tumefactive demyelinating disease, and an infection. After extensive multidisciplinary discussions, we proceeded with a stereotactic biopsy.

Several intraoperative specimens were obtained via stereotactic biopsy. Preliminary results were consistent with a high-grade glioma due to central necrosis and surrounding hypercellular brain tissue. Thus, the decision was made to convert to a craniotomy and resect the mass in its entirety. Intraoperatively, the mass appeared firm and rubbery. It was locally invasive into the dura; however, the dural attachments were easily detached with well-defined planes circumferentially.

Postoperative MRI demonstrated a gross total resection of the enhancing mass, with the continued presence of bifrontal white matter hyperintensities. Postoperative labs were notable for a WBC of 3.6 K/MM3. The patient had no postoperative complications and was discharged home on postoperative day five.

Forty-eight hours after discharge, the initial acid-fast staining was positive and raised concern for a Mycobacterium tuberculosis infection. An immunodeficiency panel revealed positivity for HIV, and she was subsequently diagnosed with acquired immunodeficiency syndrome (AIDS). The patient disclosed that her deceased husband was an intravenous drug user and was previously diagnosed with HIV. She was started on anti-mycobacterial therapy. Antiretroviral therapy was deferred due to concerns for immune reconstitution syndrome in the setting of mycobacterial infection. The final pathology was consistent with the diagnosis of Mycobacterium genavense.

Pathology results

**Figure 3:**
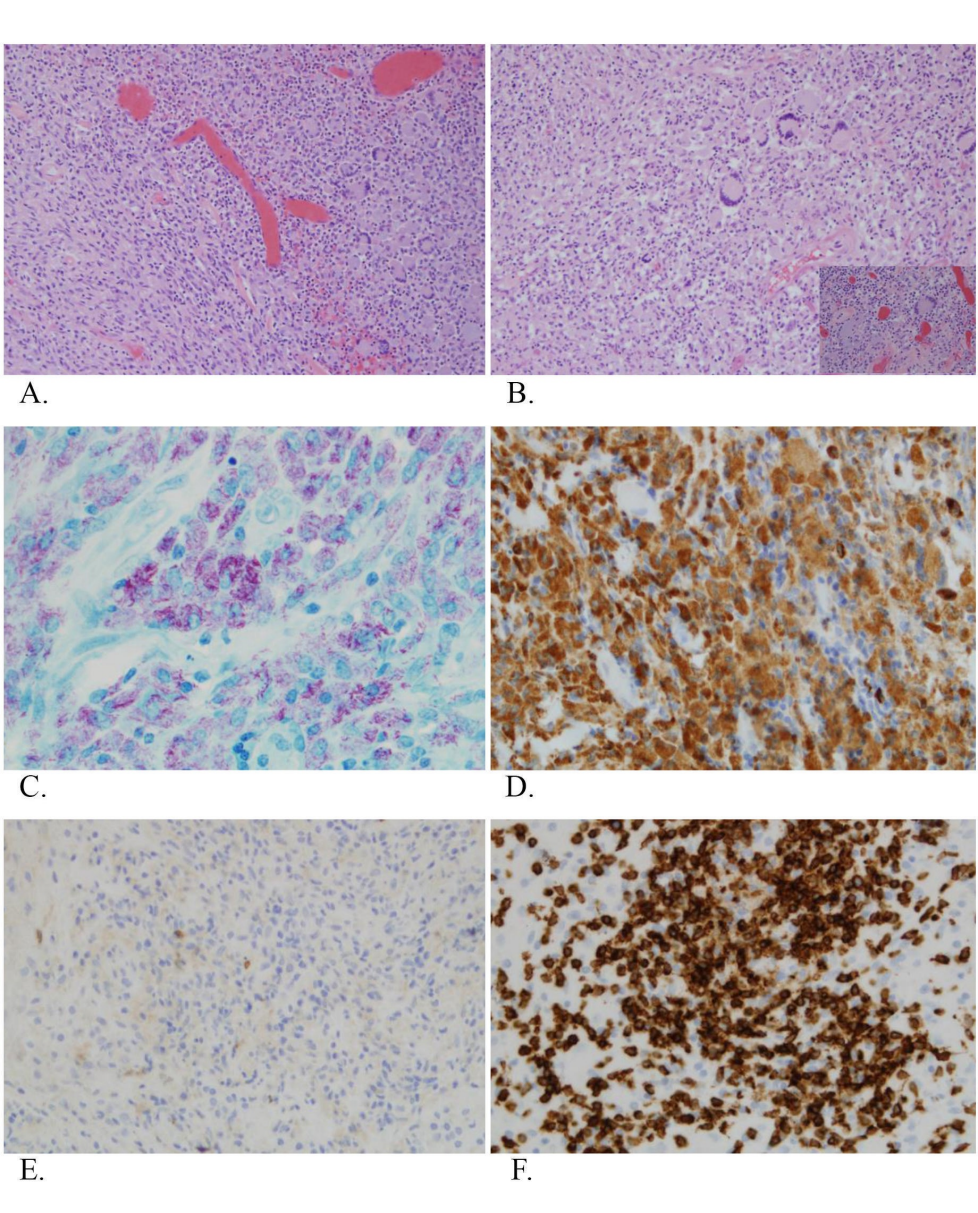
Histologic sections A, B demonstrate hematoxylin and eosin (H&E) staining of a collection of round and spindle shaped cells with scattered multinucleated giant cells (100x, 200x magnification, respectively); B: Inset B demonstrates light blue content within mononucleated and multinucleated cells (400x magnification); C: Acid-fast staining demonstrates numerous red rods within lesional cells (400x magnification), strongly expressing CD 68 immunostaining; D: 200x magnification); E, F: Lymphocyte-rich area show predominance of CD8 positive cells (F, 200x magnification) with absence of CD4 positive cells (E, 200x magnification) likely corresponding to patient’s HIV positive status. HIV: human immunodeficiency virus

On histologic sections, the sample was composed of spindle-shaped and epithelioid histiocytes with abundant granular cytoplasm and strongly positive staining for CD68 (Figure 3A-3B). The cytoplasm of these histiocytes was packed with small acid-fast bacilli (AFB) (Figure 3C-3D). A sharp demarcation was present between the lesion and normal adjacent brain parenchyma. The surrounding tissue demonstrated reactive astrocytosis and dense lymphocytic infiltrates surrounding the nearby blood vessels. Focal necrosis and patchy collection of chronic inflammatory cells were observed. CD8 positive cells predominated over CD4 expressing cells, likely as a result of the patient’s HIV-positive status (Figure 3E-3F). Moreover, scattered multinucleated giant cells, microabscesses, and leptomeningeal involvement were present.

Given the positive AFB staining in the lesion, a polymerase chain reaction (PCR) analysis was performed, revealing Mycobacterium genavense deoxyribonucleic acid (DNA) with a 16S ribosomal ribonucleic acid (rRNA) gene primer set. The PCR was negative for both Mycobacterium tuberculosis and avium.

## Discussion

Non-tuberculous mycobacterium (NTM) are increasingly recognized as human pathogens [2]. Several categories of NTMs exist, distinguished by bacterial growth rate and pigment production. The most relevant of these organisms in immunocompromised patients are M. genavense and the M. avium complex (MAC).

The Mycobacterium avium complex is the most common NTM and causes pulmonary infections in immunocompetent patients [3]. In immunocompromised patients, MAC infections typically disseminate, presenting with fevers, night sweats, weight loss, and abdominal pain [2, 4]. In contrast, M. genavense infections are rare within the NTM family. They are a slow-growing mycobacterium found in tap water, pets, and in the gastrointestinal tract of healthy individuals [5].

The pattern of Mycobacterium genavense infection presents with predominantly digestive rather than respiratory symptoms. When disseminated, M. genavense spreads to the bone marrow, liver, small intestines, spleen, and lymph nodes [6]. Visualization with Ziehl-Neelsen staining typically reveals infiltrating histiocytes packed with numerous acid-fast bacilli [6-7]. M. genavense requires a special supplementation of Mycobactin J for recovery on culture and needs to be incubated for at least eight to 12 weeks, making laboratory diagnosis challenging and slow [3].

In the original studies describing M. genavense by Bottger in 1994, nearly 10^10^ organisms were found per gram of infected tissue [6]. The inflammatory response to the bacterium is scarce and rare; necrotic foci are often observed. A foamy histiocytic reaction is described as the bacterium’s most unique feature [6]. Diagnosis is made by the histological and molecular analysis of biopsied samples as well as available cultures. The 16S rRNA gene amplification with universal primers, in addition to histological confirmation with positive Ziehl-Neelsen staining and blood or intestinal mucosal cultures, can confirm the diagnosis of M. genavense infection [7].

Symptoms of disseminated M. genavense infections are similar to those observed in MAC infections and include fever, abdominal pain, weight loss, lymphadenitis, hepatosplenomegaly, and progressive anemia [8]. M. genavense commonly affects the enteric system and is exceedingly rare as an infection of the central nervous system; only two previous cases of intracranial M. genavense have been reported. Of those two cases, both involved HIV-positive patients who presented with the insidious onset of neurological deficits [4, 9].

In the first case, the patient had a CD4 count of 84/mm^3^ at the time of hospitalization and was admitted for a grand mal seizure. On physical exam, he was found to have Kaposi’s sarcoma, mild diffuse weakness, tremors, memory impairment, and slurred speech. On MRI of the brain, a single homogeneously enhancing, left parietal mass measuring 2 cm x 1.3 cm with dural involvement was identified. The mass was later biopsied, and antibiotics for M. genavense were initiated [4].

The second case also involved a patient with the diagnosis of HIV who presented with dysarthria, aphasia, and mild right-sided hemiparesis. M. genavense was identified in thoracic and abdominal lymph nodes. The MRI of the brain demonstrated multiple enhancing subcortical masses with perilesional edema. A biopsy revealed an M. genavense infection, and antibiotics were initiated [9].

In contrast to the previous two cases, our patient had no neurologic deficits on admission and the only manifestation of her immunosuppressed state was the history of refractory chronic sinusitis and leukopenia. The diagnosis of HIV was made after brain biopsy results showed an intracranial infection with a mycobacterium species.

As mentioned previously, M. genavense infections develop mostly in severely immunocompromised patients, particularly those with advanced HIV and AIDS with CD4+ counts less than 50-100 cells per µL [1]. Thus, in this population, NTM infections with M. genavense should be considered when encountering homogeneously enhancing, dural-based intracranial lesions on MRI brain scans.

Furthermore, when faced with intracranial lesions in immunocompromised patients, it is prudent to start off with tissue biopsy to rule out infectious etiologies, as large craniotomies may lead to longer recovery periods and a higher risk of complications.

Treatment

Once the diagnosis of intracranial NTM infection is confirmed via tissue biopsy, multidrug antibiotic therapy should be initiated. Treatment of disseminated NTM infections includes clarithromycin (1,000 mg/d) or azithromycin (250 mg/d) and ethambutol (15 mg/kg/d) with or without rifabutin (150-350 mg/d) [3].

For adults with AIDS and a CD4+ count less than 50 cells/µL, prophylactic treatment should be administered via 1,200 mg/week of azithromycin or 1,000 mg/d of clarithromycin or 300 mg/day of rifabutin for opportunistic infections [3].

When treating an NTM infection in the setting of HIV and AIDs, one must be cognizant of immune reconstitution syndrome (IRS). IRS is defined as a paradoxical worsening of the clinical symptoms of an HIV-positive patient diagnosed with an infectious process following the initiation of antiretroviral therapy. The pathophysiology of this syndrome is thought to be secondary to the restoration of immunity to specific pathogens and a hyperactive immune response. NTM infections are associated with IRS and commonly result in lymphadenitis and abscess formation [10]. In cases of IRS, highly active antiretroviral therapy (HAART) may be delayed until the resolution of an active infectious process, as seen in the presented case.

The previous two reported cases of intracranial M. genavense infections were both managed successfully with the antibiotic therapy outlined above.

## Conclusions

M. genavense is a rare, non-tuberculous mycobacterium that commonly presents with infections in the liver, bone marrow, small intestines, spleen, lungs, and lymph nodes. Intracranial infection with M. genavense is exceedingly rare but may occur in severely immunocompromised patients.

Immunocompromised patients with atypical appearing, homogeneously enhancing intracranial lesions should be evaluated for M. genavense infections. Diagnosis should be confirmed via biopsy rather than gross total resection, as more extensive surgical procedures increase the risk of postoperative infection in immunocompromised patients. Treatment for M. genavense is similar to other NTM organisms using multidrug antibiotic therapy.
